# Placement of TOF-Cuff® on the lower leg for neuromuscular and blood pressure monitoring during anesthetic induction for shoulder surgeries

**DOI:** 10.1007/s00540-019-02712-7

**Published:** 2019-11-23

**Authors:** Alexander Dullenkopf, Katja Horn, Marc P. Steurer, Florian Hess, JoEllen Welter

**Affiliations:** 1 Institute for Anaesthesia and Intensive Care Medicine, Spital Thurgau Frauenfeld, Pfaffenholzstrasse 4, 8501 Frauenfeld, Switzerland; 2 Department of Anaesthesia and Perioperative Care, University of California at San Francisco, San Francisco, USA; 3 Department of Orthopedic Surgery and Traumatology, Spital Thurgau, Frauenfeld, Switzerland

**Keywords:** Neuromuscular monitoring, Accelerometry, Neuromuscular transmission

## Abstract

The aim of this study was to compare two devices for neuromuscular monitoring during anesthetic induction. TOF-Cuff^®^ was installed on the lower leg stimulating the tibial nerve, while the more conventional TOF-Scan^®^ was installed over the ulnar nerve at the wrist.

Methods

Twenty adult patients were enrolled in this prospective, controlled study. Train-of-four (TOF) was recorded every 15 s until TOF ratio of 0%. Mean arterial blood pressure (MAP) was assessed with TOF-Cuff® and with standard anesthesia monitoring from the brachial artery. MAP was measured before and after anesthetic induction. Time to TOF ratio = 0% was compared with one-sample *t* test and Bland–Altman plots.

Results

Patients received 0.53 ± 0.09 mg atracurium per kg body weight intravenously. Mean time to TOF ratio = 0% was 150.8 s (± 43.7) for TOF-Scan^®^, and 174.4 s (± 42.7) for TOF-Cuff^®^ (*p* = 0.1356). Bias was − 15.9 (95% confidence interval − 37.5 to 5.6) with 95% limits of agreement of − 95.2 to 63.3. Twenty-five percent of the patients had a technical issue with a TOF-Cuff^®^ measurement. For MAP, mean difference was 1.4 (95% confidence interval − 2.4 to 5.2) with 95% limits of agreement of − 22.7 to 25.5.

Conclusion

The time from administration of a common dose of atracurium to a TOF ratio of 0% assessed with TOF-Cuff® stimulating the tibial nerve compared to TOF-Scan® stimulating the ulnar nerve showed large limits of agreement in Bland–Altman analysis. There was a high failure rate with TOF-Cuff® measurements on the lower leg.

## Introduction

Neuromuscular monitoring as part of standard monitoring during anesthetic induction has been widely recommended [1, 2], since it allows for objective quantification of the effect of neuromuscular blocking agents (NMBA). Accelerometry, which is the current clinical standard for neuromuscular monitoring [1-4], is most often conducted using electrical impulses that stimulate the ulnar nerve on the medial forearm. The resulting motor response is measured based on acceleration of the thumb, and the stimulation pattern most frequently used is the train-of-four (TOF). This pattern uses the motor responses of the 1st and 4th stimuli to calculate a percentage to indicate the level of neuromuscular blockade, with a value of 100% indicating ‘no measurable neuromuscular block’ and 0% as ‘no response to 4th stimulus’.

A newly developed neuromuscular monitor, the TOF-Cuff® (RGB medical devices; Madrid, Spain), incorporates both stimulating electrodes and sensors into a blood pressure cuff [5-7]. In addition to the ease of application and use, this dual-purpose device does not require specific limb positioning or expensive components used with other monitoring systems. Not only can the TOF-Cuff® be used to stimulate the plexus brachialis and assess the degree of the neuromuscular block from changes in pressure in the cuff, it has also explicitly been marketed for use on the lower leg (calf). In this location, the electrodes stimulate the posterior tibial nerve and the sensors assess the response of the corresponding musculature. The option to use this device in a different location on the body is particularly appealing for procedures such as arm/shoulder surgery, bilateral breast surgery or axillary lymph node resections. However, its effectiveness at assessing neuromuscular block and blood pressure at this location has not been reported in the literature.

The primary aim of this study was to compare the performance in neuromuscular monitoring of the TOF-Cuff^®^ located on the lower leg (calf) with a clinically more conventional device for accelerometry (TOF-Scan^®^; IDMed; Marseille, France) on the classic position stimulating the ulnar nerve at the forearm. A secondary aim was to compare blood pressure values obtained from the TOF-Cuff® location compared to standard non-invasive blood pressure monitoring on the upper arm (brachial artery). The primary endpoint was the time lapse from the administration of neuromuscular blocking agents to a TOF ratio of 0%. The secondary endpoint was the blood pressure readings recorded at the beginning and the end of the anesthesia induction. Our hypothesis was that these two endpoints would not vary significantly between the TOF-Cuff® and the standard device.

## Methods

This prospective, controlled study was approved by the Ethics Commission East Switzerland (EKOS; BASEC-nr. 2016-02044; 22.12.2016), registered with the German Clinical Trial Register (www.DRKS.de, DRKS00012373) and informed consent from patients was obtained prior to inclusion. While both TOF-Scan^®^ and TOF-Cuff^®^ devices were placed on the same patients, all therapeutic decisions requiring TOF or similar information were based solely on findings from our institutional standard, TOF-Scan^®^.

### Neuromuscular monitoring

Although the gold standard for neuromuscular monitoring is mechano- or electromyography, the greater practicality of quantitative accelerometry makes it the current clinical standard. The most commonly used nerve to assess the neuromuscular transmission is the ulnar nerve just proximal to the wrist. After applying an electrical current (e.g., 60 mA) via two cutaneous electrodes, the muscular response of the adductor pollicis brevis muscle can be quantified. Using the “train-of-four” stimulation pattern, a series of four stimuli were used to measure a decreasing response from the muscle in the presence of a non-depolarizing neuromuscular blockade. When all four stimuli produce a mechanical response, the train-of-four is expressed as a percentage of the last response compared to the first. Accordingly, a TOF ratio = 0% indicates less than four mechanical responses to stimulation. With increasing effect of the NMBA, fewer responses occur, ultimately resulting in no muscular contraction, which then corresponds to a TOF count of zero.

### Conduct of study

TOF-Scan^®^ was used in this study as the control device to which the investigational device, TOF-Cuff^®^, could be compared. At our institution, this standard device is pre-programmed to deliver 60 mA to stimulate the ulnar nerve. The thumb is secured in the optimal position using a special brace provided by the manufacturer. The minimum time between two TOF measurements is 15 s. Conversely, the TOF-Cuff^®^ is a dual-purpose device that acts as both a non-invasive, oscillometric, blood pressure monitor and a quantitative neuromuscular monitor offering the same stimulation patterns as TOF-Scan^®^ (including train-of-four). TOF-Cuff^®^ employs integrated electrodes within the blood pressure cuff to stimulate the brachial plexus of the upper arm or the posterior tibial nerve of the lower leg proximal to the ankle joint (institutional standard current is 40 mA). The muscular response is then measured using integrated sensors within the same cuff. The minimum time between measurements is 12 s. For the purposes of this study, the TOF-Cuff^®^ was placed on the lower leg with patients in a supine position.

### Participant selection

Consecutive adult patients undergoing shoulder surgery requiring both general anesthesia and the administration of neuromuscular blocking agents for anesthetic induction according to institutional protocols of the Cantonal Hospital of Frauenfeld (Spital Thurgau Frauenfeld, Frauenfeld, Switzerland) were assessed for inclusion in the study. We excluded patients who were emergency cases, pregnant, morbidly obese, and those who had peripheral arterial occlusive and/or neuromuscular disease, any contraindications to atracurium, or skin infections on the leg or arm. Patients who were already enrolled in another study (including previous enrolment in this study) were also excluded.

### Anesthetic induction

Standard monitoring applied to all patients (MP 30, Philips; Zurich, Switzerland) included ECG, non-invasive blood pressure monitoring (NIBP) and SpO_2_ (with NIBP and SpO_2_ being applied to the non-surgical arm), as well as modified EEG monitoring using BIS (bispectral index, BIS; Phillips; Zurich, Switzerland) before a free-flowing peripheral i.v. was inserted (also on the non-surgical arm). The TOF-Scan® was placed on the non-surgical arm using the manufacturer-supplied brace, while TOF-Cuff® was located on the lower leg after carefully preparing the skin and positioning the electrodes according to the manufacturer’s instructions just above the ankle joint.

Patients were pre-oxygenated in supine position and received 1.5 µg/kg fentanyl prior to induction with propofol-based TCI (target-controlled infusion; Schnider TCI model; Alaris PK Infusion Pump, CareFusion, Rolle, Switzerland) set to an effect compartment concentration of 6 µg/ml. The study protocol allowed for dose variations as well as the addition of 0.15 µg/kg/min of remifentanil at the discretion of the attending anesthesiologist. After BIS levels reached 60 or less, the blood pressure was measured using both the standard NIBP monitor and the TOF-Cuff^®^, and then the two neuromuscular monitors were started simultaneously. This allowed for TOF-Scan^®^ reference values (normalization) to be obtained prior to the administration of the neuromuscular blocking agent. After the establishment of a stable baseline measurement (3 times TOF = 100%), 0.5 mg/kg atracurium was administered. Once TOF-Scan^®^ indicated TOF ratio = 0%, the patient’s airway was secured. After that, blood pressure measurements were repeated as above. The anesthetic was maintained using propofol-based TCI. The above-mentioned anesthesia induction protocol corresponds to our institutional standard.

### Data collection

Both demographic data and key aspects of the anesthetic process were collected. We documented the side of the body on which the devices were placed, the laterality of the patients (left or right dominant), and the circumference of the lower leg on the upper and lower edge of the cuff of TOF-Cuff^®^. We recorded the values of the TOF measurements every 15 s. In the case that a measurement was ongoing at that time point, the value was recorded immediately after that measurement concluded. If a technical error or missing/no show of TOF values occurred, TOF-Scan^®^ electrodes, or the TOF-Cuff^®^ cuff, respectively, were replaced once after again carefully preparing the skin at the measurement site. The values from both devices were recorded until both neuromuscular monitors showed a value of TOF ratio = 0%. The number of attempts to successful tracheal intubation was noted. Blood pressure values (systolic, MAP, and diastolic) were recorded at the beginning and the end of the anesthesia induction. Technical problems relating to both devices were also documented. At the end of the anesthetic course, patients were examined for any injuries or adverse reactions to either neuromuscular monitor, which was then documented accordingly.

### Statistics

Time differences from the administration of the NMBA to TOF ratio = 0% between both neuromuscular monitors were tested with a one-sample *t* test and illustrated with a Bland–Altman plot. Individual raw data for TOF measurements were graphically displayed. Blood pressure was measured twice in each patient. Therefore, all statistical estimates for blood pressure that were based on two measurements were weighted to be 0.5. Differences in MAP measurements between TOF-Cuff^®^ and standard monitoring were also illustrated with a Bland–Altman plot and tested with a one-sample weighted *t* test. Since the distribution of some of the differences in blood pressure measurements was skewed to the left, exact Wilcoxon signed-rank tests for each of the measurements were also performed. Lin’s concordance correlation coefficient was computed for time to TOF ratio = 0% and for MAP blood pressure measurements. The frequency of technical problems occurring with both devices was compared using the Exact McNemar’s test.

According to Bland and Altman [8], when assessing agreement between two methods of clinical measurement, the sample size depends on the chosen accuracy level of the limits of agreement. The formula to calculate the 95% confidence interval of the limits of agreement is defined as: ± 1.96 root (3/n)s, where *s* is the standard deviation of the differences between measurements by the two methods and *n* is the sample size. Thus, a sample size of 20 gives a 95% confidence interval of the limits of agreement of ± 0.76 s.

For all comparisons, *p* < 0.05 was the threshold to establish statistical significance. Data analysis was performed using Microsoft Excel 2010 (Microsoft, Redmond, USA), Stata version 15.1 (StataCorp, College Station, Texas, USA), and R programming language (version 3.3.3) (R Core Team 2017).

## Results

Twenty patients met the criteria to be enrolled in the study. Demographic data are presented in Table 1. Overall, TOF-Scan^®^ was installed on the left arm in 80% of the cases. TOF-Cuff^®^ was placed on the left calf in 70% of the cases. The standard adult-sized TOF-Cuff® was used for all patients. The calf circumference measurements are presented in Table 1. All patients underwent intravenous anesthetic induction using propofol and fentanyl (TCI 6.1 µg/ml (± 0.2), and 0.1 ± 0.0 mg, respectively). Forty percent of the patients also received remifentanil for anesthetic induction. A mean of 0.53 (± 0.09) mg of atracurium per kg body weight was administered intravenously.

**Table 1:** Demographic data of study patients (n = 20)

Age	Years	52.1 (± 18.3)
Gender	Male	12 (60%)
ASA physical status	I–II–III–IV	1 (5%)–16 (80%)–3 (15%)–0
Height	m	1.70 (± 0.1)
Weight	kg	79.6 (± 14.3)
Body mass index (BMI)	kg/m^2^	27.3 (± 4.0)
Dominant side	Right	18 (90%)
Lower leg circumference at proximal edge of cuff	cm	29.9 (± 3.3)
Lower leg circumference at distal edge of cuff	cm	22.2 (± 2.6)

Data displayed as mean (± SD) or *n* (%)

*ASA* American Society of Anesthesiologists

**Fig. 1:**
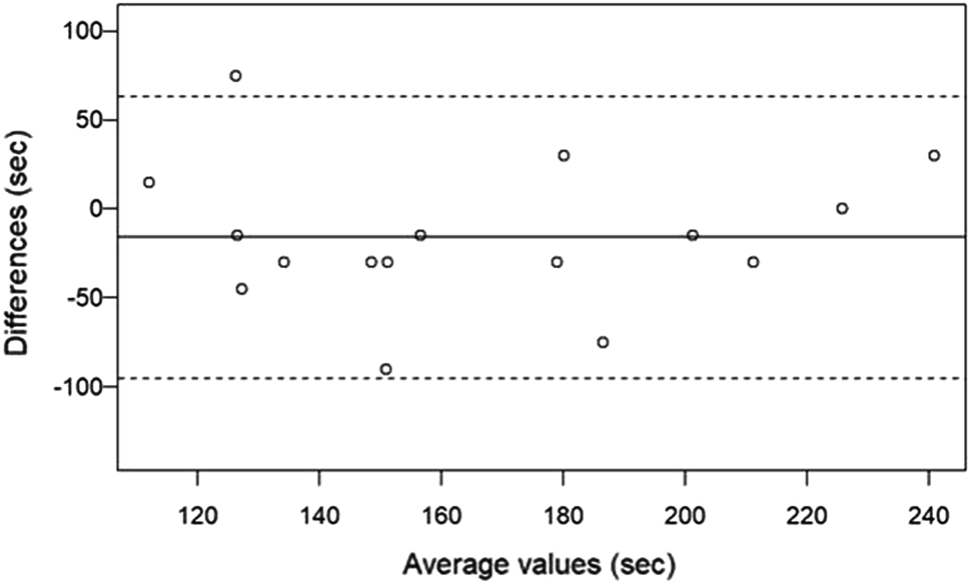
Bland–Altman plot for time to TOF ratio = 0%. The solid line illustrates the mean difference and the dashed lines indicate average difference ± 1.96 standard deviation of the difference

**Fig. 2:**
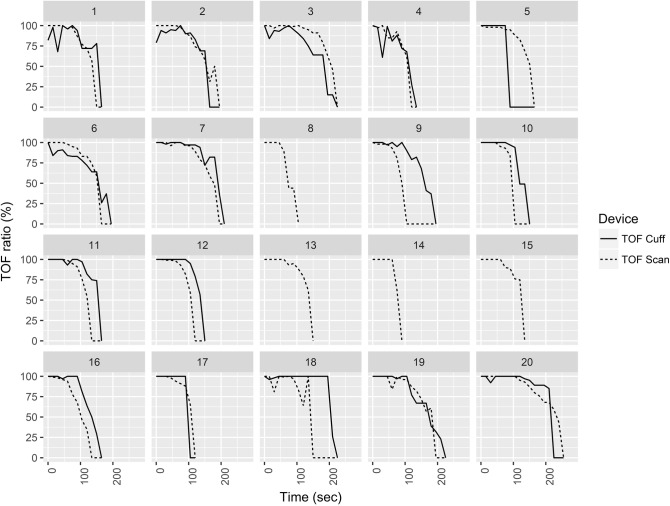
Relaxation over time for each patient for TOF-Scan^®^ and TOF-Cuff^®^

The mean time from the administration of the neuromuscular blocking agent to TOF ratio = 0% was 150.8 s (± 43.7) for TOF-Scan^®^ and 174.4 s (± 42.7) for TOF-Cuff^®^ (*p* = 0.1356). Lin’s concordance correlation coefficient was 0.53 (95% CI: 0.10–0.79). TOF-Scan^®^ indicated TOF ratio = 0% faster in 11 of 16 cases (69%; 4 missing values for TOF-Cuff^®^). Bias was − 15.9 (95% confidence interval − 37.5 to 5.6) with 95% limits of agreement of − 95.2 to 63.3. Figure 1 shows the Bland–Altman plot for TOF comparisons. Individual raw data are displayed in Fig. 2.

At least one technical issue (defined as ‘no result’ or ‘error message’) occurred in 25% of patients during the TOF-Cuff® measurements, however, no such problems occurred with TOF-Scan® measurements. In 4 patients (20%), no TOF-Cuff® measurement was obtained despite replacing the cuff. Statistically, the frequency of problematic readings did not differ significantly (*p* < 0.0625).

All patient`s tracheas were successfully intubated in the first attempt.

**Fig. 3:**
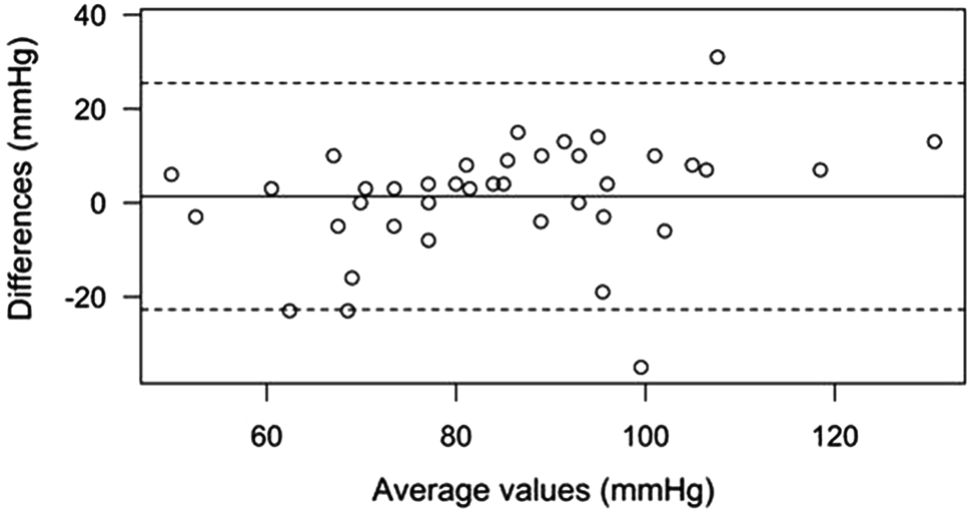
Bland–Altman plot for mean arterial (MAP) blood pressure. The solid line illustrates the weighted mean difference and the dashed lines indicate average difference ± 1.96 weighted standard deviation of the difference

The values obtained from the non-invasive blood pressure measurements are shown in Table 2. For the mean arterial blood pressure, the mean difference was 1.4 (95% confidence interval − 2.4 to 5.2) with 95% limits of agreement of − 22.7 to 25.5. The one-sample *t* test indicated that the weighted mean difference (1.4, 95% CI − 2.4 to 5.2) of the MAP between TOF-Cuff^®^ and standard monitoring was not significantly different from zero (*p* = 0.488). The *p* value for the exact Wilcoxon signed-rank test was 0.079 for the first and 0.941 for the second measurement. Lin’s concordance correlation coefficient was 0.78 (95% CI 0.63–0.87). Figure 3 shows the Bland–Altman plot for mean arterial blood pressure comparisons.

**Table 2:** Comparison of blood pressure measurement using TOF-Cuff^®^ and standard monitoring (MP 30, Philips; Zurich, Switzerland)

Time point of assessment		TOF-Cuff^®^	Standard monitoring	*P* value
Beginning of anesthesia induction	Systolic	143.0 (130.5–151.0)	133.0 (117.8–149.3)	0.178^1^
	MAP	94.0 (86.0–104.0)	89.5 (81.8–97.3)	0.079^1^
	Diastolic	63.0 (58.5–70.0)	74.5 (68.8–85.0)	< 0.001^1^
End of anesthesia induction	Systolic	107.0 (89.8–117.3)	109.0 (93.8–117.0)	0.805^1^
	MAP	72.0 (61.8–84.0)	76.5 (69.8–80.8)	0.941^1^
	Diastolic	51.5 (41.8–57.3)	64.0 (54.5–73.5)	< 0.001^1^
All	Systolic	124.6 (± 29.8)	123.0 (± 29.4)	0.616^2^
	MAP	85.5 (± 20.3)	84.2 (± 16.6)	0.488^2^
	diastolic	56.2 (± 13.5)	70.7 (± 15.5)	< 0.001^2^

Data displayed as mean (± SD) or median (IQR) in mmHg; all assessments for blood pressure that were based on two measurements are weighted by weight = 0.5

*MAP* mean arterial pressure

^1^Exact Wilcoxon signed-rank test

^2^One-sample weighted *t* test

Lastly, none of the patients showed signs of injury at relaxometry sites after the measurements.

## Discussion

This study compared the performance of the dual-purpose TOF-Cuff® with (1) a clinically widely used accelerometry neuromuscular monitor (TOF-Scan^®^) during anesthetic induction, and (2) with the clinical standard for non-invasive blood pressure monitoring at the beginning and end of anesthetic induction. The TOF-Cuff^®^ was placed on the lower leg stimulating the posterior tibial nerve and the TOF-Scan^®^ on the forearm stimulating the ulnar nerve. There were large 95% limits of agreement and a rather low correlation between the time needed for both devices to reach 0% TOF ratio. TOF-Cuff^®^ used on the leg showed a high rate of measurement failure.

Non-depolarizing neuromuscular blocking agents facilitate tracheal intubation and improve surgical conditions. They are particularly useful during orthopedic procedures on the shoulder, e.g., in spite of high-quality guidelines for the monitoring of patients receiving NMBA [1, 2], neuromuscular monitoring continues to be underutilized [9-11]. Currently available neuromuscular monitors are sometimes cumbersome and prone to erroneous readings, particularly when patient and/or surgeon positioning creates another barrier to their use [12]. In the case of shoulder surgery, all necessary monitoring installations (e.g., blood pressure measurement (possibly invasive), pulse oximetry, neuromuscular monitoring, and infusions) have to take place on the one available arm.

Another important aspect of shoulder surgery is blood pressure management for both perfusion and surgical visibility during endoscopic surgery.

In comparison to conventional neuromuscular monitors, TOF-Cuff^®^ offers the advantage of being used for measuring both neuromuscular blockade and non-invasive blood pressure, not only at the standard position (upper arm), but also on the lower leg, a location less likely to be impacted by patient and staff positioning. TOF-Scan® assesses acceleration of the thumb in multiple planes by sensors integrated in a special brace. However, it cannot be used to assess acceleration in the foot arising from stimulation of the posterior tibial nerve.

In this study, we found some clinically important differences between the measurements obtained with the two devices. The mean time to sufficient neuromuscular block (defined as TOF ratio = 0%) showed differences in individual patients of more than 60 s between the TOF-Scan^®^ and TOF-Cuff^®^. For endotracheal intubation, this finding has clinical relevance.

These differences could relate to the measuring method (accelerometry vs. assessment of pressure changes in a cuff arising from muscular activity) or to the site of assessment. It is well known that certain muscles (e.g., diaphragm) are relatively more resistant to neuromuscular blocking agents than the more delicate musculature of the hand (adductor pollicis) or glottis [1, 2]. Each anatomic location or muscle (group) requires its own definition of the ideal value for endotracheal intubation. The distance from the heart and thereby the time from injection to effect of a NMBA could also play a role in this. However, the difference in time after administration of the NMBA to reaching the hand compared to reaching the lower leg most probably is not sufficient to explain variations of up to 60 s. Further, it has to be noticed that there was only a relatively small bias and negative *t* testing, indicating no systematic deviation in comparison to the large limits of agreement.

Furthermore, we encountered ‘technical problems’ in 25% and ‘no result at all’ in 20% of the patients when using TOF-Cuff® on the leg, which is an unacceptably high number. It is possible that a higher intensity of the electric current would have resulted in a more reliable stimulation of the posterior tibial nerve. Due to the conical shape of the lower leg, the mean circumference of the calf at the proximal border of the cuff was 29.9 cm (± 3.3) compared to 22.2 cm (± 2.6) at the distal border in our study population. The recommended (upper arm) circumference range for the standard adult TOF-Cuff® cuff is 27.5–36 cm. Consequently, the selected cuff size may have resulted in at least one of the two electrodes making insufficient contact.

Veiga-Ruiz et al. found that blood pressure monitoring using the TOF-Cuff^®^ at the upper arm was reliable [5]. In most cases, the blood pressure taken from the lower leg just proximal to the ankle is slightly higher than when it is taken from the upper arm, which is consistent with our findings. Systolic values of the ankle–brachial index of 0.9–1.3 are considered normal [13]. Our findings of the systolic blood pressure were well within this range. Overall, our measurements showed an acceptable agreement between the devices, with the mean arterial pressures not differing significantly.

Our study sample size was similar to other reported investigations [7, 14], but still relatively small and homogenous, which limits our ability to generalize our results. Using the gold standard in neuromuscular monitoring, mechano- or electromyography, as our control device could have strengthened the robustness of our investigation. However, TOF-Scan® is one of the few available devices for the clinical standard of accelerometry and it has been found to be an acceptable device for clinical research [14]. Lastly, the main limitation of our study regarding complete coverage of a neuromuscular monitoring can be seen in the lack of data during recovery from neuromuscular block up to the point for tracheal extubation. It is important to investigate not only “front-end kinetics” (i.e., anesthesia induction) but also “back-end kinetics” (recovery period) as well. Certainly, this needs further study as postoperative residual neuromuscular blockade presents an even more important issue than intubating conditions. We limited our study to the anesthetic induction, because after anesthetic induction our patients did not follow a strict protocol concerning NMBA re-dosing or NMBA reversal at the end of surgery.

In conclusion, the time from administration of a common dose of atracurium to a TOF ratio of 0% showed large limits of agreement when the TOF-Cuff^®^ was placed on the lower leg compared to placement of TOF-Scan^®^ on the hand. Mean arterial blood pressure measurement showed no significant difference. There was a high failure rate with TOF-Cuff^®^ measurements on the lower leg which needs further investigation.

## Conflict of interest

The authors state that there is no conflict of interest.
